# Coalescence dynamics of 3D islands on weakly-interacting substrates

**DOI:** 10.1038/s41598-020-58712-1

**Published:** 2020-02-06

**Authors:** V. Gervilla, G. A. Almyras, B. Lü, K. Sarakinos

**Affiliations:** Nanoscale Engineering Division, Department of Physics, Chemistry and Biology, Linköping University, SE 581 83 Linköping, Sweden

**Keywords:** Materials science, Theory and computation, Atomistic models, Computational methods, Thermodynamics, Surfaces, interfaces and thin films

## Abstract

We use kinetic Monte Carlo simulations and analytical modelling to study coalescence of three-dimensional (3D) nanoscale faceted silver island pairs on weakly-interacting fcc(111) substrates, with and without concurrent supply of mobile adatoms from the vapor phase. Our simulations show that for vapor flux arrival rates *F* < 1 monolayer/second (ML/s) coalescence manifests itself by one of the islands absorbing the other via sidewall facet migration. This process is mediated by nucleation and growth of two-dimensional (2D) layers on the island facets, while the supply of mobile atoms increases the nucleation probability and shortens the time required for coalescence completion. When F is increased above 1 ML/s, coalescence is predominantly governed by deposition from the vapor phase and the island pair reaches a compact shape via agglomeration. The crucial role of facets for the coalescence dynamics is further supported by a mean-field thermodynamic description of the nucleation energetics and kinetics. Our findings explain experimental results which show that two-dimensional film growth morphology on weakly-interacting substrates is promoted when the rate of island coalescence is suppressed. The present study also highlights that deviations of experimentally reported film morphological evolutions in weakly-interacting film/substrate systems from predictions based on the sintering and particle growth theories may be understood in light of the effect of deposition flux atoms on the energetics and kinetics of facet-layer nucleation during coalescence.

## Introduction

Coalescence—the process of merging two or more atomic islands or grains into a single cluster—is an ubiquitous phenomenon in material synthesis technologies, and largely determines the morphology and microstructure of metallurgical alloys^1^ and vapor-deposited thin films^2,3^, as well as the size and shape of free-standing and supported nanoparticles^4,5^. The most established description of island coalescence relies on the sintering theory, according to which shape equilibration of coalescing clusters proceeds via isotropic atomic surface diffusion^6^. This scenario is relevant for temperatures above the roughening transition limit *T*_*R*_ for which generation of ample amounts of adatom-kink pairs facilitates continuous and uniform mass transport from the convex to the concave areas of the island cluster^7^. However, many material synthesis processes, including vapor-based growth of thin films and supported nanoparticles on weakly-interacting substrates, occur below *T*_*R*_. At these conditions, generation of adatom kink-pairs is infrequent, leading to formation of three-dimensional (3D) islands bounded by flat sidewall facets^8^. Combe *et. al*.^9^ studied shape equilibration of single free-standing faceted 3D nanoparticles upon annealing at temperatures smaller than *T*_*R*_ showing that mass transport is mediated by nucleation and growth of two-dimensional (2D) layers on the facet surfaces. The relevance of this mechanism for coalescence of 3D clusters supported on a substrate has not been investigated. Moreover, a key aspect of coalescence during film growth, i.e., the constant supply of mobile atoms from the vapor flux and the way by which this affects the microscopic processes controlling island reshaping, remains to be understood.

In the present work, we employ kinetic Monte-Carlo (KMC) simulations and thermodynamic modelling, to: (i) study merging of nanoscale 3D silver (Ag) faceted island pairs supported on weakly-interacting fcc(111) substrates with and without concurrent vapor deposition; and (ii) understand the kinetics and energetics of the atomic-scale processes that drive shape equilibration under the influence of an external source of mobile atoms emanating from a deposition flux. We find that faceted island pairs coalesce by one of the islands absorbing the other, whereby mass transport is mediated by repeated nucleation and growth of layers on the facet surface. We also find that vapor-atom deposition rates below one monolayer (ML)/s promote facet nucleation; whereas for rates above one ML/s inhibit this process and formation of compact cluster shapes occurs via agglomeration. Our results are consistent with experimental observations in weakly-interacting film/substrate systems which show that the tendency toward 3D film morphological evolution is suppressed when island coalescence is inhibited by increasing the deposition rate. Moreover, our findings show that the effect of deposition flux on coalescence dynamics may be relevant for understanding disparities between experimental data for the scaling behavior of the percolation transition thickness during film growth on weakly-interacting substrates and predictions based on the sintering and droplet growth theories^2,6,10–18^. The overall results of this study provide insights for enabling controlled growth of metal films and supported nanostructures on weakly-interacting substrates^19^, including oxides and 2D materials, which is a key step in fabrication of high-performance nanoelectronic, optoelectronic, catalytic and sensing devices^5,20–22^.

## Results and Discussion

### KMC simulations

We perform simulations of island pair coalescence for sizes in the range N = 200–1400 atoms per island, at temperatures *T*_*s*_ = 500K and 800K and vapor arrival rates *F* between 0 and 50 ML/s. More details on the KMC algorithm and the simulation methodology are provided in the "Methods" section. The latter section also provides detailed illustrations and descriptions of the geometrical and crystallographic features encountered during coalescence.

**Figure 1:**
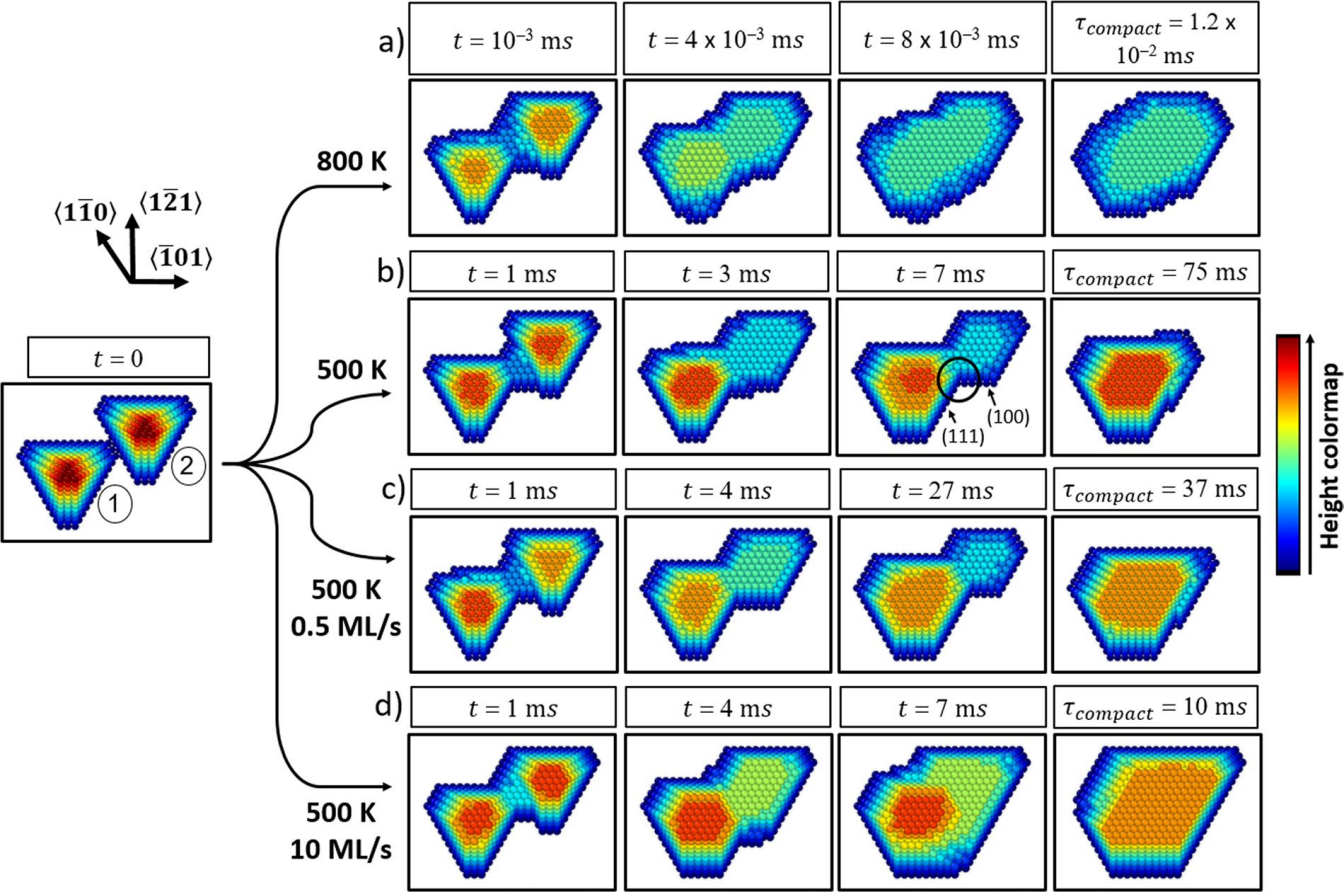
Simulated changes undergone by a cluster upon physical contact of two faceted 3D islands (700 atoms/island), at temperatures of 800 K in (**a**) and 500 K in (**b**,**d**). In (**c**,**d**), relaxation proceeds with a concurrent deposition flux of 0.5 ML/s and 10 ML/s, respectively. The circle at t= 7 ms in (**b**) indicates an example of an (111)/(100) facet intersection formed at the cluster neck.

Figure 1 shows simulated snapshots corresponding to different stages (i.e., time t) of shape evolution during coalescence of island pairs with a size *N* = 700 atoms per island at *T*_*s*_ = 800 *K* (*F* = 0; Fig. 1(a)), *T*_*s*_ = 500*K* (*F* = 0; Fig.1(b)), *T*_*s*_ = 500 *K* and *F* = 0.5 *ML*/*s* (Fig. 1(c)), and *T*_*s*_ = 500 *K* and *F* = 10 *ML*/*s* (Fig. 1(d)). The initial snapshot in Fig. 1 (*t* = 0 *s*) is the common starting point for all four displayed simulation series, and all the boxes have the same size at all times and simulated conditions.

For *T*_*s*_ = 800 *K* (Fig. 1(a)), the island surface becomes rough, hosting a large density of kink sites, most notably for *t* > 4 × 10^−3^ *ms*. The simulations show that atoms detach from kinks at the island tops and diffuse to occupy the highly-coordinated sites adjacent to the intersection of the islands to form a neck (see Supplementary Video 1). Subsequent re-shaping proceeds by further atomic migration toward available kink sites in the neck area until the cluster becomes compact, in a similar fashion to shape relaxation of rough spheres^10,11^.

Decrease of *T*_*s*_ to 500 K (Fig. 1(b)) allows the initial flat sidewall facet structure of the islands to be maintained, which leads to the formation of a sharp facet-facet intersection in the neck area (*t* = 3 *ms*). From this observation we conclude that *T*_*s*_ = 500 *K* corresponds to a temperature below the roughening transition temperature *T*_*R*_ for our simulated systems, while *T*_*s*_ = 800 *K* is equal or larger than *T*_*R*_. The estimated KMC *T*_*R*_ range is consistent with experimental measurements showing that for Ag *T*_*R*_ ≲ 0.4 ⋅ *T*_*melt*_^23,24^, where *T*_*melt*_ = 1235 *K*. After neck formation at *T*_*s*_ = 500 *K*, the cluster undergoes a relaxation through a different pathway than at *T*_*s*_ = 800 *K*, in which island (2) (right top) is absorbed by island (1) (bottom left) via repeated facet migration (see Supplementary Video 2). Detailed inspection of simulation output shows that this process is initiated by detachment of atoms from kink sites on island (2). These atoms then cross the neck toward island (1) and get incorporated into growing sidewall layers. Evaluation of simulation data shows that there is a net atomic flux *j*_*net*_=2.13 atoms/ms from island (2) to (1) after neck formation. The explanation for this anisotropy in the direction of atomic migration lies in the cluster geometry. The neck between the two islands is formed by the intersection of (111) facets on island (1) and (100) facets on island (2) (see circled area in Fig. 1(b), also in “Methods” section). Thus, an atom encounters a different local atomic environment when attempting to diffuse toward the two sides of the intersection. This is reflected on the bond counting scheme used for calculating process barriers in the KMC algorithm^25^, according to which an atom has to overcome an energy barrier ${E}_{A}^{intersection-(100)}=0.61$ eV for detaching from the intersection to island (2) vs. a barrier of ${E}_{A}^{intersection-(111)}=0.57$ eV for intersection-island (1) detachment.

In their study of freestanding nanoparticle equilibration, Combe *et al*.^9^ showed that the growth of a new layer on a smooth facet of particles undergoing reshaping requires the formation of a 2D critical nucleus by aggregation of diffusing atoms. This mechanism is also relevant in our case; an atom that crosses the neck of a coalescing cluster either aggregates with another atom or finds a highly-coordinated attachment site on a newly formed 2D nucleus on island (1). In the opposite case, the atom returns to island (2). The lower kink density, i.e., the lower density of stable adatom attachment sites on the surface of the faceted islands at *T*_*s*_ = 500 K, compared to the rough islands at 800 K, yields a considerably lower effective mass transport rate and can explain the three orders of mangitude longer equilibration time at 500 K (75 ms) compared to 800 K (1.2 × 10^−2^ *ms*).

The presence of a continuous deposition flux with a rate *F* = 0.5 *ML*/*s* (Fig. 1(c)) does not qualitatively change the stages of shape evolution, compared to Fig. 1(b). However, the cluster shape evolves faster and a compact shape is formed at time *τ*_*compact*_ = 37 *ms* vs. *τ*_*compact*_ = 75 *ms* without deposition flux. This is because atoms arriving at the substrate from the vapor phase diffuse toward the coalescing cluster and ascend onto the cluster facets^26^, yielding a lager density of mobile atoms relative to the case of island annealing (*F* = 0). This, in turn, enhances the probability of 2D facet nucleation, and together with the net flux of atoms from island (2) to island (1) over the neck area discussed previously, accelerates the cluster equilibration.

For a deposition rate *F* = 10 *ML*/*s* and temperature *T*_*s*_ = 500 *K* (Fig. 1(d)), the island cluster reaches a compact shape after *τ*_*compact*_ = 10 *ms* by following a different pathway, compared to Fig. 1(b,c) : deposited atoms attach with equal probabilities on both sides of the concave section of the cluster, while no facet migration from island (2) to island (1) occurs, and island absorption is not observed (see Supplementary Video 3). Hence, the evolution dynamics of the cluster is, in this case, dictated by the adatom arrival rate, and, as effectively no mass redistribution takes place while the cluster becomes compact, we refer to the merging of the islands as agglomeration instead of coalescence.

**Figure 2:**
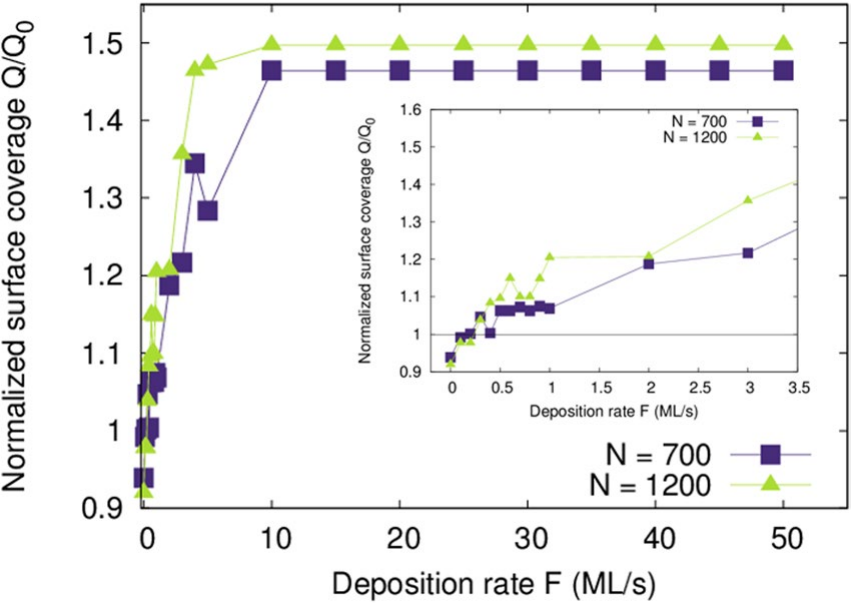
Normalized surface coverage *Q*/*Q*_0_ of a coalescing cluster when a compact shape is reached, as a function of deposition rate F, for N = 700 and 1200 atoms/island, at T = 500 K (extracted from KMC simulations). The inset shows a zoom in the F range 0 to 3.5 ML/s.

In order to explore the correlation among the coalescence mechanisms discussed in Fig. 1 and reported experimental data on film morphological evolution as a function of deposition conditions, we extract the substrate surface coverage Q from our simulations at the time *τ*_*compact*_ when the cluster reaches a compact shape (see “Methods” section for details on the calculation of the surface coverage Q and *τ*_*compact*_). Figure 2 presents values of Q, normalized to the area covered by the cluster at t = 0 (*Q*_0_), as a function of F, for island sizes N = 700 and 1200 atoms/island, at T = 500 K. For deposition rates *F* ≤ 0.5 *ML*/*s* (see inset in Fig. 2) the normalized surface coverage exhibits values *Q*/*Q*_0_ ≤ 1. Further increase of F causes *Q*/*Q*_0_ to increase monotonically, until it saturates to a value 1.5 for *F* ≥ 10 *ML*/*s* (Fig. 2)).

The fact that *Q*/*Q*_0_ ≤ 1 for *F* ≤ 0.5 *ML*/*s* means that the coalescing cluster contracts upon reshaping; this is because atoms that are initially in contact with the substrate are redistributed on the sidewall facets when island (2) is absorbed by island (1). The cluster contraction is consistent with the experimentally-observed island area depletion during film growth on weakly-interacting substrates at conditions which favor island coalescence^12,13^ and lead to a pronounced 3D morphology and surface roughness built up^2,12–18,27^. Conversely, the *Q*/*Q*_0_ values in Fig. 2 being larger than 1 for *F* > 0.5 *ML*/*s* imply that the cluster expands upon island impingement and shape equilibration, since material redistribution is suppressed. This behavior is consistent with experimental results showing that conditions which suppress island coalescence favor 2D morphology and formation of smooth surfaces^2,12–18,27^.

### Energetics and kinetics of facet nucleation

**Figure 3:**
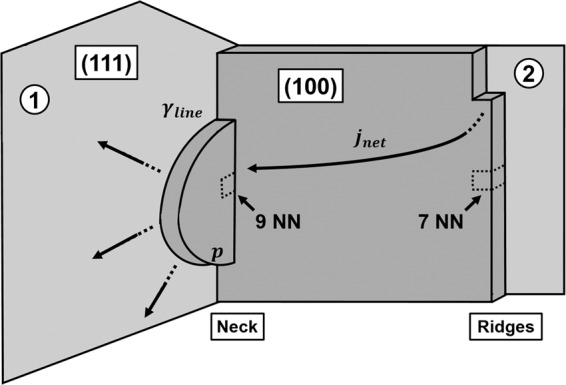
Schematic illustration of a facet migration event from island (2) to island (1). Atoms detaching from the (100) facet ridge in island (2) diffuse to the neck area, forming a nucleus (represented by a half-disc) consisting of p atoms on the (111) facet of island (1). The driving force for atomic migration with a net flux *j*_*net*_ is the increase in the number of nearest-neighbors (NN) from 7NN to 9NN. Formation of the nucleus is associated with an energy expenditure due to the formation of an edge characterized by a line tension *γ*_*line*_.

In order to gain further insights into the importance of the mechanisms established by the KMC simulations, we use thermodynamic arguments for describing the energetics of processes that drive nucleation of 2D layers on the sidewall facets at the intersection between island (1) and (2) (Fig. 1). As explained earlier in the manuscript, coalescence below the roughening transition temperature *T*_*R*_ proceeds by successive facet migration events; atoms detach from ridges in the facets of island (2), diffuse across the cluster neck and form a nucleus in the neck area of island (1), which grows by continuous adatom incorporation (see Fig. 3 for a schematic illustration and “Methods” section for representation of geometrical features). The formation of a stable semicircular 2D nucleus with a radius *r* —comprised of *p* = *πr*^2^/2Ω atoms, where Ω is the area occupied by an atom on the fcc(111) surface—is associated with an energy expenditure Δ*G*_*expenditure*_ = *γ*_*line*_*l* to create an interface (edge) of length *l* = *πr* and line tension *γ*_*line*_. Concurrently, the nucleation is driven by a decrease of the chemical potential Δ*μ* for moving atoms from the ridges to the neck, which corresponds to an energy gain of Δ*G*_*gain*_ = *p*Δ*μ*. The total free energy change due to nucleation then reads

1 $$\Delta G={\gamma }_{line}\sqrt{2\pi \Omega p}-p\left|\Delta \mu \right|,$$

from which we can calculate the nucleation barrier ΔG^*^ as

2 $$\Delta {G}^{\ast }=\frac{\pi \Omega {\gamma }_{line}^{2}}{2| \Delta \mu | }.$$

The nucleation barrier is connected^7^ to the mean nucleus formation time *τ*_*nuc*_ (i.e. the time between successive nucleation events) by the expression

3 $${\tau }_{nuc}\propto exp\left(\frac{\Delta {G}^{\ast }}{{k}_{B}T}\right).$$

To estimate ΔG^*^, we approximate *γ*_*line*_ to the value of the interatomic bond strength used for *E*_*b*_( = 0.2517 *eV*) in our KMC algorithm^25^, divided by the unit length, i.e., ${\gamma }_{line}\approx {E}_{b}/\sqrt{\Omega }$. In the absence of a vapor flux F (i.e., annealing), Δ*μ* is estimated by calculating the change in nearest-neighbor (NN) number that an atom undergoes when moves from a ridge to the neck, assuming that the islands are sufficiently large, such that moving an atom from one island to the other does not change the cluster morphology significantly. An atom in a ridge has in total 7NN, while it has 9NN in the neck area, i.e., this process is associated with an increase of 2 in the NN number (see Fig. 3) and hence |Δ*μ*| = 0.5034 *eV*. The chemical potential of a rough cluster is size/curvature-dependent since a rough surface features a uniform distribution of adatom attachment sites^6^. However, this is not the case for our coalescing faceted clusters. There atoms can only attach at kinks, at the ridges, and/or at the edges of growing facet layers, while they diffuse rapidly on flat facets. This local atomic geometry does not change with size and hence we consider Δ*μ* size-independent. By substituting the value of |Δ*μ*| and *γ*_*line*_ in Eq. 2 we obtain a ΔG^*^ = 0.1976 *eV* for nucleation upon annealing conditions.

Upon formation of a 2D nucleus on the sidewall facet, adatoms diffusing over the cluster surface can be depicted as the physical manifestation of a supersaturated 3D gas exerting a pressure

4 $${P}_{F=0}^{3D}={P}_{0}\ \left[1+exp\left(\frac{| \Delta \mu | }{{k}_{B}T}\right)\right]$$

on a 2D vapor/facet interface^7^. In Eq. 4, *P*_0_ represents the equilibrium pressure of the vapor-cluster interface; for Ag fcc(111) at 500 *K*, *P*_0_ = 9.8305 × 10^−10^ *Pa*^28^, which yields ${P}_{F=0}^{3D}=1.1540\times 1{0}^{-4}\ Pa$. For *F* > 0, the total pressure of the 3D gas increases by

5 $${P}_{F > 0}^{3D}=F\sqrt{2\pi m{k}_{B}T},$$

as vapor condensation contributes to the adatom population on the cluster surface. Hence, in this thermodynamic representation, $\overline{\Delta \mu }$ becomes the total supersaturation at the vapor/facet interface due to the combined 3D gas pressures for F = 0 and *F* > 0, i.e,

6 $$\overline{\Delta \mu }={k}_{B}T{\rm{ln}}\left(\frac{{P}_{F > 0}^{3D}+{P}_{F=0}^{3D}}{{P}_{0}}\right)$$

Assuming that all deposited atoms get incorporated to the island, the energy barrier for nucleation ΔG^*^ (Eq. 2) and the nucleus formation time *τ*_*nuc*_ (Eq. 3) can be calculated as a function of F by using Eqs. 4, 5 and 6.

**Figure 4:**
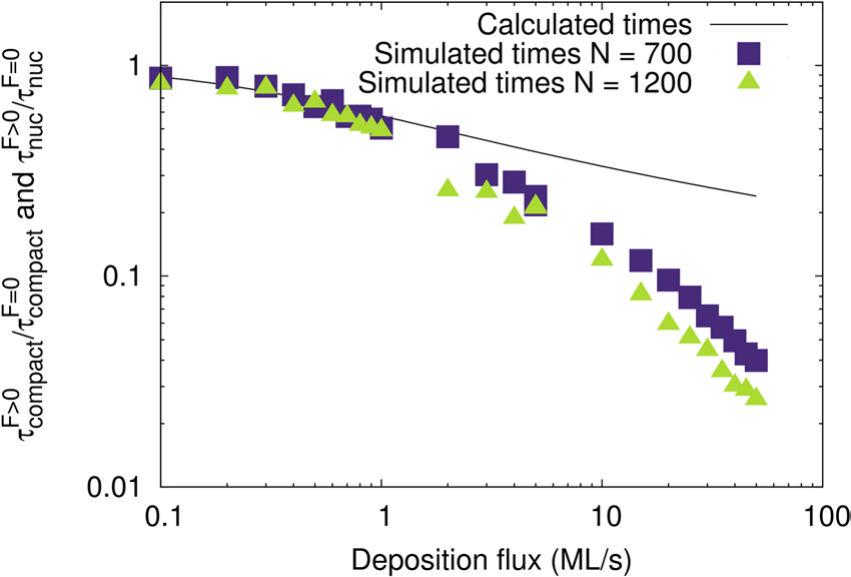
Calculated ${\tau }_{nuc}^{F > 0}/{\tau }_{nuc}^{F=0}$ from Eqs. 3, 4 and 5 (solid line) and simulated ${\tau }_{compact}^{F > 0}/{\tau }_{compact}^{F=0}$ (solid squares and triangles) ratios as a function of deposition rate F for various island sizes.

To explore the consistency among KMC simulations and thermodynamics, we use Eq. 3 to calculate the ratio ${\tau }_{nuc}^{F > 0}/{\tau }_{nuc}^{F=0}$ as a function of F. The results are plotted in Fig. 4 (solid line), along with simulated ${\tau }_{compact}^{F > 0}/{\tau }_{compact}^{F=0}$ vs. F data for cluster sizes N = 700 and 1200 atoms/island. The very good qualitative agreement between the theoretical curve and simulated data for *F* ≤ 1 *ML*/*s* underscores that: (i) coalescence at these conditions indeed proceeds by repeated cycles of facet nucleation and growth; and (ii) supply of mobile atoms from the deposition flux lowers the barrier for facet-layer nucleation and, thereby, accelerates coalescence. As F increases above 1 ML/s, the simulated data deviate increasingly from the theoretical curve, confirming that the cluster does no longer rely on facet migration to reach a compact shape, but rather on the rate at which deposited atoms fill the neck area; hence ${\tau }_{compact}^{F > 0}$ acquires a qualitatively different functional dependence on the deposition rate.

The sintering theory predicts that the time required for the shape equilibration of particles and clusters scales as ~*R*^4^ where R is the cluster radius^6^. Combining this scaling dependency with the droplet growth theory^13,29^, Carrey and Maurice^30,31^ showed that the nominal film thickness at which a film that grows in 3D fashion (e.g., during metal film deposition on weakly-interacting substrates) reaches percolation *θ*_*perc*_ scales with the vapor arrival rate as *θ*_*perc*_~ *F*^−1/3^. Experimentally, the scaling law *θ*_*perc*_ ~ *F*^−1/3^ is only partially validated with reported scaling exponents ranging between −0.33 and −0.25^12,14,17,27^. It has been suggested that the disparity between experiments and theory is the result of islands being faceted, which leads to equilibration times scaling as ~ *R*^*a*^, with *a* > 0. The results of the present study show that the dynamics of coalescence are more complex and possible effects of mobile atom supply should be taken into account when trying to explain scaling behaviors of film morphology as a function of growth conditions.

## Summary and Outlook

We performed KMC simulations of coalescence of faceted 3D Ag island pairs supported on a weakly-interacting substrate, at temperatures *T*_*s*_ = 800 K and *T*_*s*_ = 500 K, and for vapor flux arrival rates F in the range 0 to 50 ML/s. At *T*_*s*_ = 800 K and without deposition, simulations show that island surface is rough and coalescence proceeds via isotropic surface diffusion. In contrast, for *T*_*s*_ = 500 K islands remain faceted and mass transport relies on nucleation of new layers on the facets leading to one island being absorbed by the other via facet migration. The addition of mobile atoms from the vapor phase with *F* < 1 *ML*/*s* enhances nucleation probability and accelerates the coalescence. By increasing F above 1ML/s, the process of facet layer growth is predominantly governed by deposition of material from the vapor phase so that the island cluster reaches a compact shape via agglomeration. The existence of two regimes, i.e., coalescence vs. agglomeration is further supported by an analytical thermodynamic description of facet nucleation energetics and dynamics.

Our findings explain experimental results which show a transition from 3D to 2D film growth morphology on weakly-interacting substrates when rate of coalescence is suppressed, as well as the origin of changes in thin film roughness and grain boundary number densities when varying the magnitude of vapor flux arrival rate^2,12–18^. In addition, our results point out that disagreement between theoretical works predicting different scaling behavior for the percolation transition thickness than that observed experimentally might be due to the effect of deposition rate in the coalescence of island pairs. In this way, the results from this study may become relevant for knowledge-based synthesis of thin films and nanostructures with controlled morphology on weakly-interacting substrates^8^, in particular on 2D-materials for nanoelectronic, sensing, and catalytic devices^5,20,21^.

## Methods

KMC simulations^32,33^ are performed using our previously-developed code^25^, which has been validated for homoepitaxial Ag/Ag(111) growth and was used to model atom-by-atom formation and shape evolution of individual Ag islands on fcc (111) weakly-interacting substrates. Process rates for the KMC algorithm are calculated via Arrhenius equations ${\nu }_{i}={\nu }_{0}{e}^{(-{E}_{A}^{i}/{k}_{B}T)}$, where *ν*_0_ = 10^13^ *s*^−1^ is the Debye frequency of the substrate, and ${E}_{A}^{i}$ the activation barrier of the diffusion jump *i*. We compute ${E}_{A}^{i}$ as a function of the change in the nearest- and next-nearest neighbors, that an atom undergoes as it moves from its initial to its final adsorption site^25^. The substrate is a rhombus with 100 atoms per side, while periodic boundary conditions are used. Hence one monolayer (ML) corresponds to 10^4^ atoms. We implement the weakly-interacting substrate by explicitly lowering the pairwise atom-atom bond strength between island and substrate atoms to one half of the bond strength between two island atoms. More details on the implementation of the KMC algorithm can be found in ref. ^25^.

**Figure 5:**
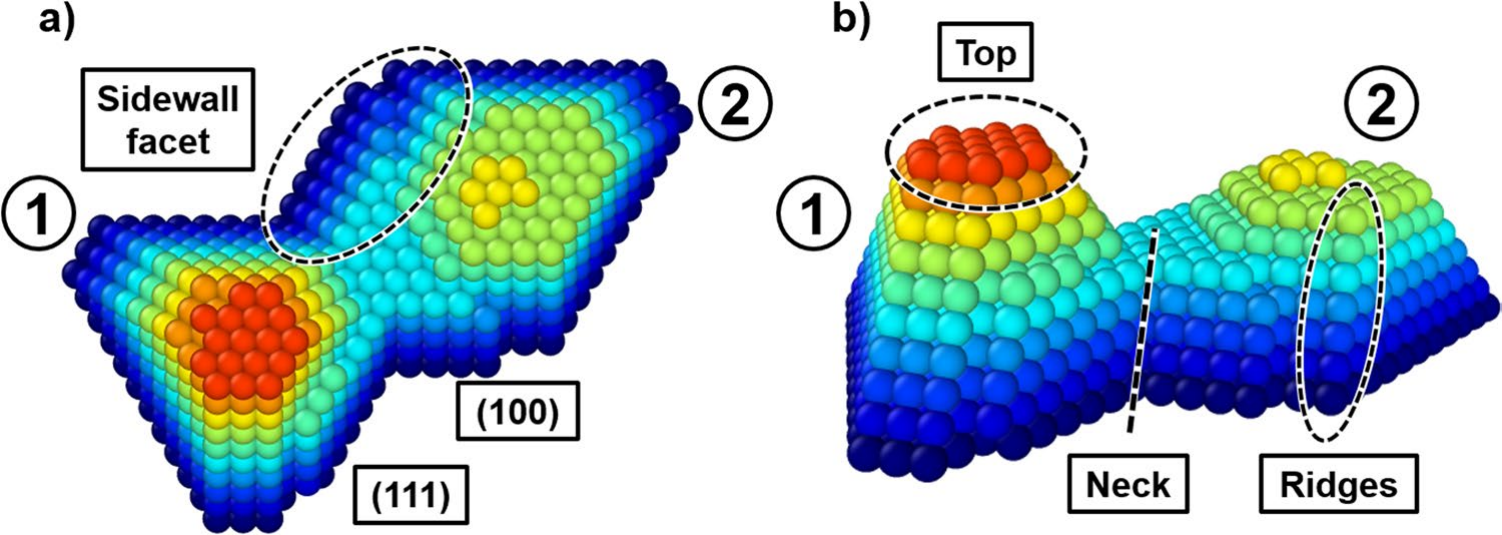
Snapshot of a coalescing cluster, taken from the top (**a**) and from the side (**b**) of the simulation box, displaying all relevant crystallographic and geometrical features.

We perform simulations of coalescence during annealing of island pairs at temperatures *T*_*s*_ = 500 *K* and 800 *K*, whereby the number of atoms per island *N* ranges from 200 to 1400. The chosen island sizes represent a low end of possible sizes that can be encountered in experiments^34^. Nonetheless, we argue that this choice does not impair our ability to draw reliable conclusions for coalescence of islands with sizes beyond the simulated ones because: (i) islands exhibit a self-similar shape evolution^25^, which means that the atomic scale processes that govern coalescence are size-independent; and (ii) formation and coalescence of faceted islands has been reported to occur over a wide range of sizes, spanning from nanometers to micrometers^34^. For the same *N* range, we also simulate coalescence of island pairs for *T*_*s*_ = 500 *K* at the presence of a continuous deposition flux with rates *F* = 0.1–50 *ML*/*s*. The simulations show that for any deposition rate at least ~100 atoms are added to the simulated system from the vapor phase, which provides sufficient statistics for drawing reliable conclusions. Moreover, for each of the aforementioned simulation settings, we perform 25 statistically independent runs. Each data point presented in Figs. 2 and 4 represents the mean over all values extracted from the simulations. Initial island shapes are pyramids with a height-to-radius aspect ratio of ~1.7, bounded predominantly by (111) smooth sidewall facets, in accordance with island shapes obtained in our previous computational study^25^ and experimental data on vapor-based growth of noble-metal nanostructures^35–37^. The island centers are aligned along the (110) direction (see Figs. 1 and 5). This orientation represents a typical scenario of two islands impinging on each other, as islands tend to align their crystallographic axes upon contact^38^. Figure 5 shows a top-view (Fig. 5(a)) and a side-view (Fig. 5(b)) snapshot of a coalescencing island cluster and highlights key geometrical and crystallographic features used and discussed throughout the manuscript.

**Figure 6:**
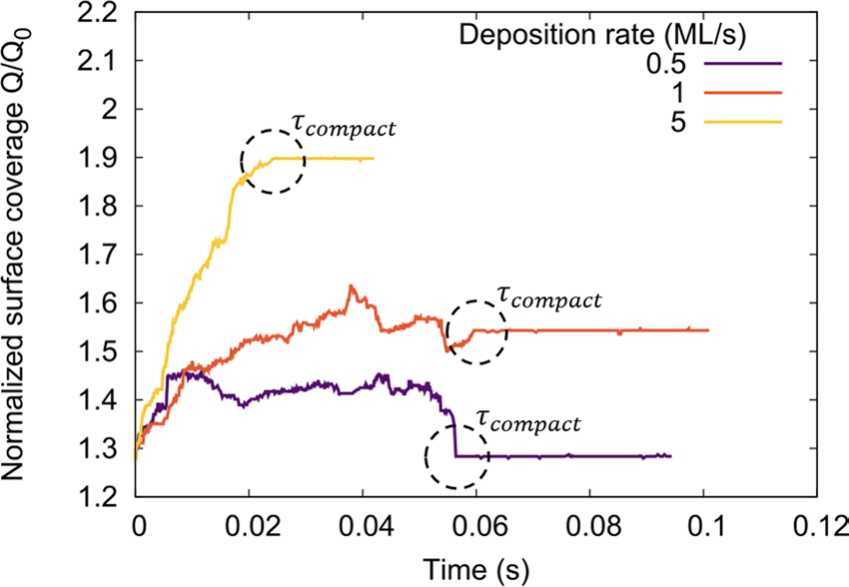
Evolution of the normalized surface coverage *Q*/*Q*_0_ of a coalescing cluster with N = 700 atoms/island as a function of time, for different deposition rates. The time *τ*_*compact*_ at which the cluster becomes compact is marked with a dashed circle in each of the cases.

We study the coalescence process by visualizing simulations output and tracking the temporal evolution of the substrate surface coverage Q, i.e., the area of the substrate covered by the cluster (Fig. 6). We determine the time *τ*_*compact*_ at which the cluster reaches a compact shape—a cluster is considered compact at the point beyond which the concave facet-facet intersections disappear—and we find that Q saturates to a steady-state value at *τ*_*compact*_. Note that at the time at which *Q*/*Q*_0_ reaches steady state, the cluster attains a compact shape, and it reaches its maximum in-plane size, deposited atoms predominantly contribute to top-layer nucleation. Eventually, nucleation of new sidewall facets occurs as more material is deposited resulting in an increase of *Q*/*Q*_0_ (not shown in Fig. 6). Simulations are visualized using the Ovito freeware^39^.

## Supplementary information

Supplementary Video 1

Supplementary Video 2

Supplementary Video 3

## Data Availability

The source code for the simulations can be obtained by mailing the corresponding author.
